# Supplementary material for: Potassium channel TASK-5 forms functional heterodimers with TASK-1 and TASK-3 to break its silence
Source: Nat Commun. 2024 Aug 30;15:7548. doi: 10.1038/s41467-024-51288-8 (PMC11364637; doi:10.1038/s41467-024-51288-8)
Supplement: Supplementary file 1 — Supplementary Information [file 41467_2024_51288_MOESM1_ESM.pdf]

Supplementary Information

Supplementary Figure 1

a

```
TASK-1 MKRQNVRTLALIVCTFTYLLVGAAVFDALESEPELIERQRLELRQQELRARYNLSQGGYEELERVVLRLKPHKAGVQWRF80
TASK-5 MRRPSVRAAGLVLCTLCYLLVGAAVFDALESEAESGRQRLLVQKRGALRRKFGFSAEDYRELERLALQAEPHRAGRQWKF80

TASK-1 AGSFYFAITVITTIGYGHAAPSTDGGKVFCMFYALLGIPLTLVMFQSLGERINTLVRYLLHRAKKGLGMRRADVSMANMV160
TASK-5 PGSFYFAITVITTIGYGHAAPGTDSGKVFCMFYALLGIPLTLVTFQSLGERLNAVVRRLLLAAKCCLGLRWTCVSTENLV160

TASK-1 LIGFFSCISTLCIGAAAFSHYEHWTFFQAYYYCFITLTTIGFGDYVALQKDQALQTQPQYVAFSFVYILTGLTVIGAFLN240
TASK-5 VAGLLACAATLALGAVAFSHFEGWTFFHAYYYCFITLTTIGFGDFVALQSGEALQRKLPYVAFSFLYILLGLTVIGAFLN240

TASK-1 LVVLRFMTMNAEDEKRDAEHRALLTRNGQAGGGGGGGSAHTTDTASSTAAAGGGGFRNVYAEVLHFQSMCSCLWYKSREK320
TASK-5 LVVLRFLVASADWPERAA----------RPPSPRPPGAPESRGLWLPRRPARSVGSASVFCHV-HKLERCA------RDN303

TASK-1 LQYSIPMIIPRDLSTSDTCVEQSHSSPG--GGGRYSDTPSRRCLCSGAPRSAISSVSTGLHSLSTFRGLMKRRSSV394
TASK-5 LGFSPP------------------SSPGVVRGGQ-------------APRPGA------------------RWKSI330
```

b

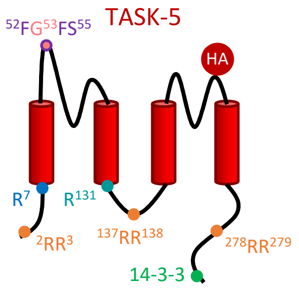

Supplementary Fig. 1| TASK-5 resists to form homomeric channels at the plasma membrane. a, Sequence alignment of human TASK-1 (NM_002246.3) and TASK-5 (AF294350.1). ^{2}RR^{3}, ^{137}RR^{138}, ^{278}RR^{279} (orange): putative di-arginine signals; R7 or R131 (blue), putative "latch" residues of the "X-gate"; G53 (light red): site at which the G53C dimerization mutation was introduced and glycosylation site in TASK-1 but not TASK-5; ^{52}FGFS^{55} (violet): difference in sequence between TASK-5 and TASK-1 mutated to get a more TASK-1 like cap structure; ^{326}RWKSI^{330} (green): putative 14-3-3 binding site; L^{214} (red): position at which the hemagglutinin (HA)-tag was introduced in TASK-1, TASK-3 or TASK-5. **b**, Cartoon of a TASK-5 subunit, illustrating the location of amino acid mutations, potential trafficking signals and the localization of the extracellular HA-epitope that was introduced. Transmembrane domains are highlighted in red and the highly conserved GYG/GFG motifs within the pore signature sequence of potassium channels are underlined.

Supplementary Figure 2

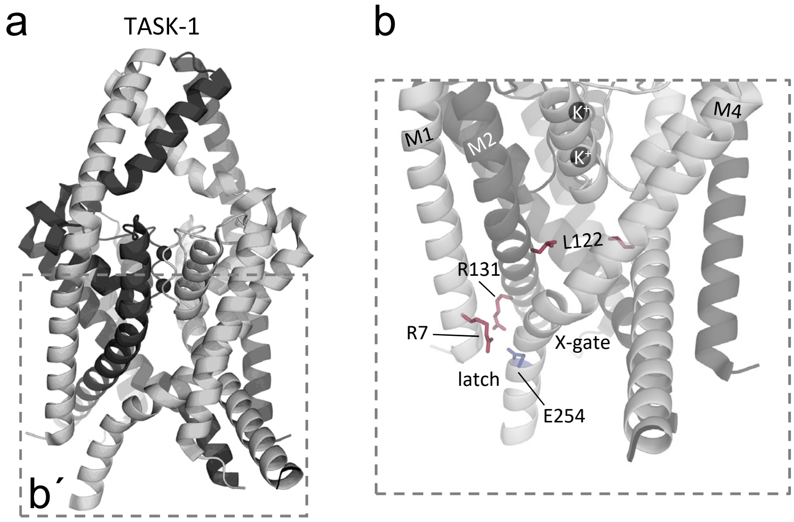

Supplementary Fig. 2| Illustration of key residues of the "latch" region involved in stabilization of the "X-gate" structure and drug binding. a, Side-view on the TASK-1 crystal structure (PDB code: 6RV2). **b,** Zoom-in to illustrate the key residues of the "latch" region involved in "X-gate" stabilization (R7, R131, R254) and drug binding in the central cavity (L122).

Supplementary Figure 3

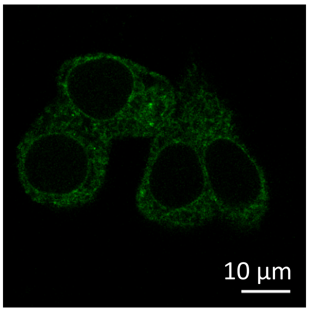

Supplementary Fig. 3| Intracellular localization of heterologously expressed homomeric TASK-5 channels. Confocal fluorescence images of HEK293T cells 48 h after transfection with TASK-5 pEGFP. Source data are provided as a Source Data file.

Supplementary Figure 4

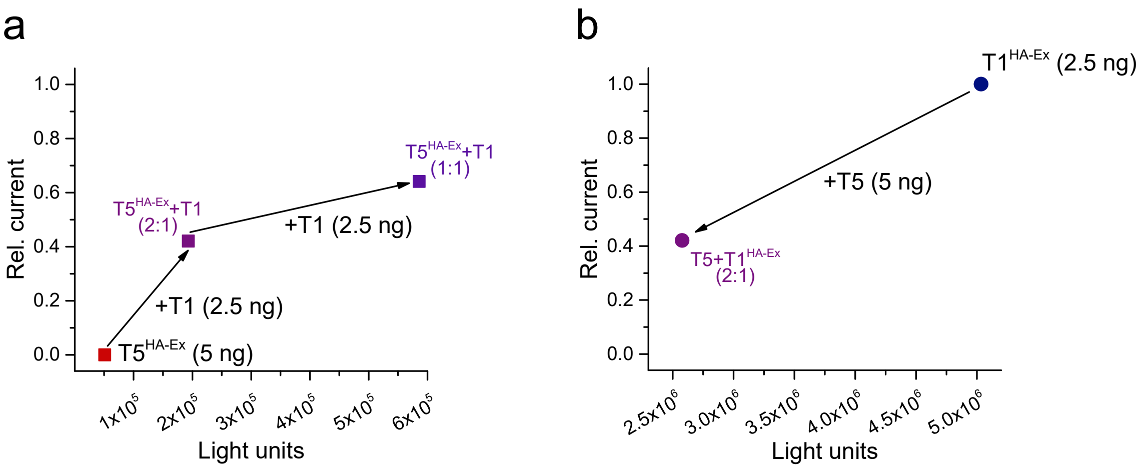

Supplementary Fig. 4| TASK-1 increases TASK-5 surface expression, while TASK-5 decreases TASK-1 surface expression. Plots of the surface expression versus current relationships for **a,** TASK-5$^{HA\text{-}Ex}$ (red) and **b,** TASK-1$^{HA\text{-}Ex}$ (blue) constructs. Surface expression levels were corrected by subtracting the background signal of non-injected oocytes.

Supplementary Figure 5

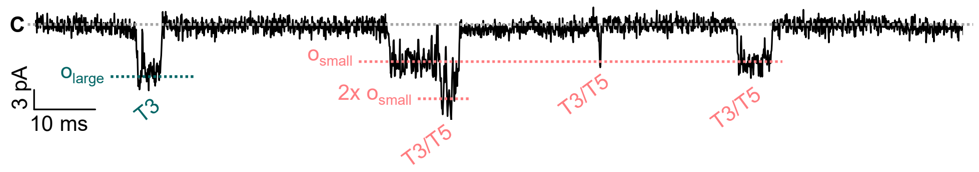

Supplementary Fig. 5| Cumulative opening events of heteromeric channels. Representative single-channel patch-clamp recording depicts opening events of TASK-3 (T3, large opening = o_{large}) and heteromeric TASK-3/5 (T3/T5, small opening = o_{small}) channels. 2x o_{small} illustrates the presence of two heteromeric channels opening simultaneously.

Supplementary Figure 6

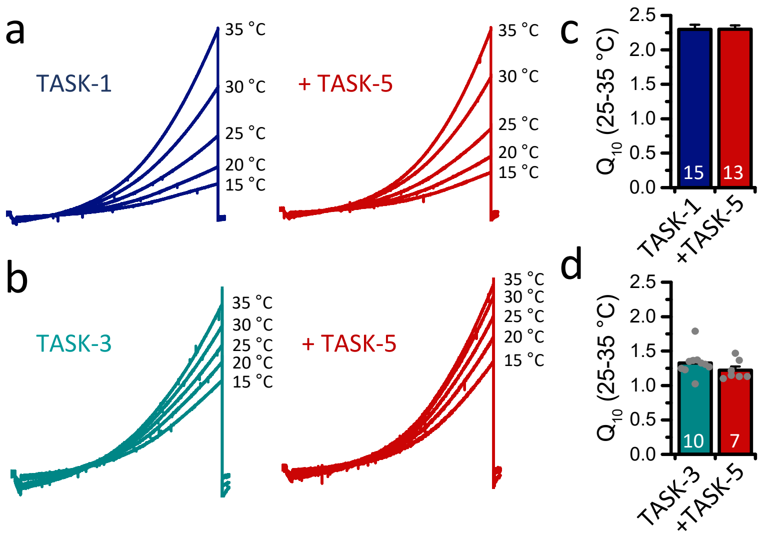

Supplementary Fig. 6| Temperature-sensitivity of TASK-5-containing TASK heterodimers. a, Representative current traces of TASK-1 (blue) and heteromeric TASK-1/TASK-5 channels (red) or **b**, TASK-3 (green) and heteromeric TASK-3/TASK-5 channels (red) at temperatures between 15 °C and 35 °C. Currents were recorded by a voltage-ramp from -120 mV to +45 mV. Q_{10} values were analyzed for **c**, homomeric TASK-1 or heteromeric TASK-1/TASK-5 and **d**, TASK-3 or heteromeric TASK-3/TASK-5. Numbers of experiments are given within the bar graphs. Data are presented as mean ± s.e.m.. Source data are provided as a Source Data file.

Supplementary Figure 7

a BAY1000493

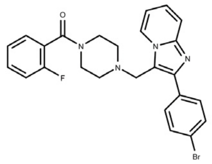

b BAY2586116

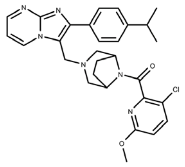

c A293

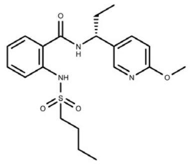

d A1899

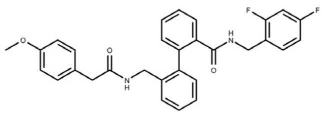

e

	TASK-1		TASK-1 + TASK-5		TASK-1 + TASK-5^{L122A}	
Substance	IC_{50} (nM)	Hill coeff.	IC_{50} (nM)	Hill coeff.	IC_{50} (nM)	Hill coeff.
BAY1000493	19.66 ± 0.14	0.68 ± 0.003	2.64 ± 0.30	0.67 ± 0.05	54.31 ± 0.62	0.61 ± 0.01
BAY2586116	27.55 ± 5.66	0.68 ± 0.08	1.04 ± 0.30	0.58 ± 0.09	0.66 ± 0.35	0.60 ± 0.19
A293 (AVE1231)	85.69 ± 13.44	0.76 ± 0.08	6.62 ± 3.13	0.55 ± 0.15	185.11 ± 29.99	0.60 ± 0.05
A1899	18.40 ± 3.95	0.68 ± 0.09	27.36 ± 1.63	0.77 ± 0.03	18.40 ± 3.95	0.69 ± 0.09

Supplementary Fig. 7| Drug-sensitivity of TASK-1 alone or TASK-1 co-expressed with TASK-5 or TASK-5^{L122A}. Chemical structures of **a**, BAY1000493, **b**, BAY2586116, **c**, A293 (=AVE1231) or **d**, A1899. **e**, IC_{50} values and Hill coefficients for the different substances tested on TASK-1 alone or TASK-1 co-expressed with TASK-5 or TASK-5^{L122A}. Data are presented as mean ± s.e.m..

Supplementary Figure 8

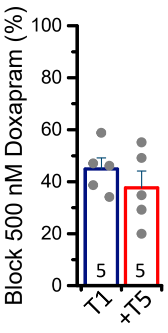

Supplementary Fig. 8| Doxapram-sensitivity of TASK-5 containing heterodimers. Block of TASK-1 (blue) or heteromeric TASK-1/TASK-5 channels (red) by 500 nM doxapram, analyzed at +40 mV. Numbers of biological replicates are given within the bar graph. Data are presented as mean ± s.e.m.. Source data are provided as a Source Data file.
