# Supplementary material for: Potassium channel TASK-5 forms functional heterodimers with TASK-1 and TASK-3 to break its silence
Source: Nat Commun. 2024 Aug 30;15:7548. doi: 10.1038/s41467-024-51288-8 (PMC11364637; doi:10.1038/s41467-024-51288-8)
Supplement: Supplementary file 3 — Reporting Summary [file 41467_2024_51288_MOESM3_ESM.pdf]

nature portfolio

Corresponding author(s): Niels Decher

Last updated by author(s): 15.07.2024

Reporting Summary

Nature Portfolio wishes to improve the reproducibility of the work that we publish. This form provides structure for consistency and transparency in reporting. For further information on Nature Portfolio policies, see our Editorial Policies and the Editorial Policy Checklist.

Statistics

For all statistical analyses, confirm that the following items are present in the figure legend, table legend, main text, or Methods section.

n/a	Confirmed	
☐	☒	The exact sample size (*n*) for each experimental group/condition, given as a discrete number and unit of measurement
☐	☒	A statement on whether measurements were taken from distinct samples or whether the same sample was measured repeatedly
☐	☒	The statistical test(s) used AND whether they are one- or two-sided<br>*Only common tests should be described solely by name; describe more complex techniques in the Methods section.*
☒	☐	A description of all covariates tested
☐	☒	A description of any assumptions or corrections, such as tests of normality and adjustment for multiple comparisons
☐	☒	A full description of the statistical parameters including central tendency (e.g. means) or other basic estimates (e.g. regression coefficient) AND variation (e.g. standard deviation) or associated estimates of uncertainty (e.g. confidence intervals)
☐	☒	For null hypothesis testing, the test statistic (e.g. *F*, *t*, *r*) with confidence intervals, effect sizes, degrees of freedom and *P* value noted<br>*Give P values as exact values whenever suitable.*
☒	☐	For Bayesian analysis, information on the choice of priors and Markov chain Monte Carlo settings
☒	☐	For hierarchical and complex designs, identification of the appropriate level for tests and full reporting of outcomes
☒	☐	Estimates of effect sizes (e.g. Cohen's *d*, Pearson's *r*), indicating how they were calculated

Our web collection on statistics for biologists contains articles on many of the points above.

Software and code

Policy information about availability of computer code

Data collection	For the two electrode voltage clamp and single channel measurements, data were digitalised with an Digidata1400A interface (Molecular Devices) and obtained via pClamp10 software (Molecular Devices). Light emission data for chemiluminescence assay was collected using GloMax luminometer (Promega).
Data analysis	Figures were prepared using OriginPro (version 2016), MarvinSketch (version 15.8.3) and Powerpoint (version 2013).<br><br>Data were analyzed with ClampFit (version 10), Excel (version 2013), Origin (version 2016) and PyMOL (version 1.3).

For manuscripts utilizing custom algorithms or software that are central to the research but not yet described in published literature, software must be made available to editors and reviewers. We strongly encourage code deposition in a community repository (e.g. GitHub). See the Nature Portfolio guidelines for submitting code & software for further information.

Data

Policy information about availability of data

All manuscripts must include a data availability statement. This statement should provide the following information, where applicable:

- Accession codes, unique identifiers, or web links for publicly available datasets
- A description of any restrictions on data availability
- For clinical datasets or third party data, please ensure that the statement adheres to our policy

The authors declare that the data supporting the findings of this study are available within the paper and its supplementary information files. Source data are provided with this paper. No original code was reported in this study.

Research involving human participants, their data, or biological material

Policy information about studies with human participants or human data. See also policy information about sex, gender (identity/presentation), and sexual orientation and race, ethnicity and racism.

Reporting on sex and gender	no human participants or human data
Reporting on race, ethnicity, or other socially relevant groupings	no human participants or human data
Population characteristics	no human participants or human data
Recruitment	no human participants or human data
Ethics oversight	no human participants or human data

Note that full information on the approval of the study protocol must also be provided in the manuscript.

Field-specific reporting

Please select the one below that is the best fit for your research. If you are not sure, read the appropriate sections before making your selection.

☒ Life sciences ☐ Behavioural & social sciences ☐ Ecological, evolutionary & environmental sciences

For a reference copy of the document with all sections, see nature.com/documents/nr-reporting-summary-flat.pdf

Life sciences study design

All studies must disclose on these points even when the disclosure is negative.

Sample size	No statistical methods were used to predetermine sample sizes. Required experimental sample sizes were estimated based on previous established protocols in the field. The sample sizes were adequate as the differences between experimental groups were reproducible.
Data exclusions	No exclusion criteria were pre-established. No data was excluded from the electrophysiology assays.
Replication	All attempts to replicate the experiments were successful. Number of biological replicates (n) are illustrated in the respective figure legends or graphs.
Randomization	This is not relevant for our study because we were not comparing two sets of individuals who had been selected as control or sample groups.
Blinding	As we were not comparing animal or human populations with individuals assigned to groups, it was not relevant to do double blind tests.

Reporting for specific materials, systems and methods

We require information from authors about some types of materials, experimental systems and methods used in many studies. Here, indicate whether each material, system or method listed is relevant to your study. If you are not sure if a list item applies to your research, read the appropriate section before selecting a response.

Materials & experimental systems

n/a	Involved in the study
	[x] Antibodies
	[x] Eukaryotic cell lines
[x]	[ ] Palaeontology and archaeology
	[x] Animals and other organisms
[x]	[ ] Clinical data
[x]	[ ] Dual use research of concern
[x]	[ ] Plants

Methods

n/a	Involved in the study
[x]	[ ] ChIP-seq
[x]	[ ] Flow cytometry
[x]	[ ] MRI-based neuroimaging

Antibodies

Antibodies used	Primary antibody:<br>Anti HA high affinity, rat monoclonal Antibody, 11867423001, Roche, dilution 1:100, Lot 34502100<br>Secondary antibody:<br>Goat anti rat peroxidase, 112-036-062, Dianova, dilution 1:500, Lot 78598
Validation	The rat monoclonal anti-HA High Affinity antibody is commonly used in the field. References can be found here:<br>https://doi.org/10.1113/jphysiol.2008.164756<br>https://doi.org/10.1111/j.1600-0854.2007.00593.x

Eukaryotic cell lines

Policy information about cell lines and Sex and Gender in Research

Cell line source(s)	HEK293T cells were commercially purchased from the Leibniz Institute ("Deutsche Sammlung für Mikroorganismen und Zellkulturen" (DSMZ)).
Authentication	HEK293T cells are purchased from DSMZ. These cell lines undergo quality control before dispatch. Cells were passaged a limited number of times before a new batch from the manufacturer was employed. Cells were monitored by regular visual inspection.
Mycoplasma contamination	The cell lines were tested for mycoplasma contamination routinely.
Commonly misidentified lines<br>(See ICLAC register)	No commonly misidentified cell lines were used.

Animals and other research organisms

Policy information about studies involving animals; ARRIVE guidelines recommended for reporting animal research, and Sex and Gender in Research

Laboratory animals	Adult, sexually mature, female Xenopus laevis toads. (Nasco®, Fort Atkinson, USA)
Wild animals	No wild animals were involved.
Reporting on sex	Xenopus laevis oocytes were utilized as a heterologous expression system in this study, and the isolation of oocytes is by nature only possible from female frogs. Nevertheless, the resulting data is transferrable to both sexes, considering that the function of the expressed ion channels and their direct pharmacological modulation are not sex specific.
Field-collected samples	No field-collected samples were used.
Ethics oversight	Xenopus laevis experiments were approved by the local ethics commission of the Regierungspräsidium Gießen (protocol code V54-19c 20 15 h 02 MR 20/28 Nr.A 23/2017, approved on 12.02.2018). All animal housing and husbandry were performed under standard conditions in accordance with institutional (Philipps University of Marburg) and national ethical and animal welfare guidelines approved by the ethics committee for animal experiments at the Regierungspräsidium Gießen, Germany. All animal procedures performed conformed to the guidelines from Directive 2010/63/EU of the European Parliament on the protection of animals used for scientific purposes and the current NIH guidelines.

Note that full information on the approval of the study protocol must also be provided in the manuscript.

Plants

Seed stocks	no plant data
Novel plant genotypes	no plant data
Authentication	no plant data
